# Age- and sex-specific reference values of biventricular strain and strain rate derived from a large cohort of healthy Chinese adults: a cardiovascular magnetic resonance feature tracking study

**DOI:** 10.1186/s12968-022-00881-1

**Published:** 2022-11-21

**Authors:** Gengxiao Li, Zhen Zhang, Yiyuan Gao, Chengcheng Zhu, Shanshan Zhou, Lizhen Cao, Zhiwei Zhao, Jun Zhao, Karen Ordovas, Mingwu Lou, Kuncheng Li, Gerald M. Pohost

**Affiliations:** 1 Shenzhen Clinical Medical College, Guangzhou University of Chinese Medicine, Shenzhen, Guangdong China; 2 The Third People’s Hospital of Longgang District, Shenzhen, China; 3 Department of Radiology, University of Washington, Seattle, USA; 4 Department of Radiology and Nuclear Medicine, Xuanwu Hospital, Capital Medical University, Beijing, China; 5 Zhouxin Medical Imaging and Healthy Screening Centre, Xiamen, China; 6 Keck School of Medicine, University of Southern California, Los Angeles, CA USA

**Keywords:** Cardiovascular magnetic resonance feature tracking, Reference value, Chinese adults, Left ventricle, Right ventricle

## Abstract

**Background:**

As a noninvasive tool, myocardial deformation imaging may facilitate the early detection of cardiac dysfunction. However, normal reference ranges of myocardial strain and strain rate (SR) based on large-scale East Asian populations are still lacking. This study aimed to provide reference values of left ventricular (LV) and right ventricular (RV) strain and SR based on a large cohort of healthy Chinese adults using cardiovascular magnetic resonance (CMR) feature tracking (FT).

**Methods:**

Five hundred and sixty-six healthy Chinese adults (55.1% men) free of hypertension, diabetes, and obesity were included. On cine CMR, biventricular global radial, circumferential, and longitudinal strain (GRS, GCS, and GLS), and the peak radial, circumferential, and longitudinal systolic, and diastolic SRs (PSSRR, PSSRC, PSSRL, PDSRR, PDSRC, and PDSRL), and regional radial and circumferential strain at the basal, mid-cavity, and apical levels were measured. Associations of global and regional biventricular deformation indices with age and sex were investigated.

**Results:**

Women demonstrated greater magnitudes of LV GRS (37.6 ± 6.1% vs. 32.1 ± 5.3%), GCS (− 20.7 ± 1.9% vs. − 18.8 ± 1.9%), GLS (− 17.8 ± 1.8% vs. − 15.6 ± 1.8%), RV GRS (25.1 ± 7.8% vs. 22.1 ± 6.7%), GCS (− 14.4 ± 3.6% vs. − 13.2 ± 3.2%), GLS (− 22.4 ± 5.2% vs. − 20.2 ± 4.6%), and biventricular peak systolic and diastolic SR in all three coordinate directions (all *P* < 0.05). For the LV, aging was associated with increasing amplitudes of GRS, GCS, and decreasing amplitudes of PDSRR, PDSRC, PDSRL (all *P* < 0.05). For the RV, aging was associated with an increase in the magnitudes of GRS, GCS, GLS, PSSRR, PSSRC, PSSRL, and a decrease in the magnitude of PDSRR, PDSRC (all *P* < 0.05). Biventricular radial and circumferential strain measurements at the basal, mid-cavity, and apical levels were all significantly related to age and sex in both sexes (all *P* < 0.05).

**Conclusions:**

We provide age- and sex-specific normal values of biventricular strain and SR based on a large sample of healthy Chinese adults with a broad age range. These results may be served as a reference standard for cardiac function assessment, especially for the Chinese population.

**Supplementary Information:**

The online version contains supplementary material available at 10.1186/s12968-022-00881-1.

## Introduction

Cardiovascular disease has long been the leading cause of morbidity and mortality in both developed and developing countries and has triggered a substantial amount of medical expenditure [1]. At present, the objective assessment of cardiac function is still one of the most critical routine tasks in cardiology. Compared with ejection fraction (EF), the most commonly used index for measuring cardiac systolic performance, myocardial deformation parameters such as strain and strain rate (SR) allow the assessment of both global and regional myocardial function and have been increasingly recognized as more sensitive indicators of cardiac insufficiency [2, 3]. In recent years, there is growing evidence that assessing cardiac function via myocardial deformation imaging is of great importance in distinguishing among various cardiac diseases and can provide important prognostic information [4–8]. Although echocardiography has long been considered the first choice for quantifying myocardial deformation, it has some inherent limitations, including a narrow acoustic window, low image spatial resolution, operator dependence, etc. [9, 10]. Cardiovascular magnetic resonance (CMR) feature tracking (FT), a promising tool for assessing myocardial mechanics, does not require additional sequences or complex postprocessing and has been well validated against the gold standard tagged CMR [11–13].

Determining cardiac reference values is essential for distinguishing normal and abnormal conditions, risk stratification, and prognosis evaluation in clinical practice. It has been well established that there are significant racial differences in the myocardial deformation measurements derived from various imaging modalities [14–16], highlighting the considerable need to establish reference values of cardiac deformation based on different ethnicities. Unfortunately, to our knowledge, there are few data on CMR-FT reference ranges for myocardial strain and SR in East Asian populations, and they are usually based on small or modest sample sizes (no more than 150 individuals) [17–19]. In addition, although many studies have explored the associations of myocardial deformation metrics with common demographic factors such as age and sex, the conclusions remain highly controversial. Therefore, the purpose of this study was to systematically provide age- and sex-specific reference values of left ventricular (LV) and right ventricular (RV) strain and SR based on a large sample of healthy Chinese adults and to further explore the correlation of these measurements with age and sex.

## Methods

### Study population

Our study population has been described previously [20]. All the subjects came from the local medical imaging and health screening institution, which provides high-end screening services for eligible community residents, with cardiovascular imaging as one of the key programs. From January 1, 2013, to June 1, 2020, a total of 1164 consecutive Chinese adults at this institution completed the dedicated screening package, including baseline characteristics, comprehensive physical examination, electrocardiogram (ECG), customized laboratory and biochemical tests (including fasting glucose and lipid tests, thyroid, liver, and kidney function tests, etc.), and whole-body imaging examinations (including echocardiography, chest X-ray or computed tomography scan, CMR, and magnetic resonance scan of the brain, spine, and abdomen). Subjects in this population, who met the following criteria were excluded: (i) a known history or overt symptoms of cardiovascular diseases; (ii) an abnormal ECG (defined as ST-T abnormalities), or abnormal findings of cardiovascular diseases such as ischemic heart diseases (e.g., wall motion abnormalities), hypertrophic cardiomyopathy (defined as end-diastolic LV wall thickness ≥ 15 mm in any segment [21]), congenital heart diseases, valvular heart diseases (defined as observed signal void jet), and cardiac tumor, etc.; (iii) known diseases that may affect cardiac morphology and function, such as anemia, hyperthyroidism, stroke, and major lung, liver, and kidney diseases; (iv) hypertension (defined as resting measured systolic blood pressure [SBP]/diastolic blood pressure [DBP] ≥ 140/90 mmHg or use of antihypertensive medication according to the seventh Joint National Committee recommendation [22]), diabetes (defined as fasting blood glucose [FBG] ≥ 7.0 mmol/L or a history of hypoglycemic medication use [23]), or obesity (defined as body mass index [BMI] ≥ 28 kg/m^2^ for the Chinese population [24]); and (v) subjects with images that were ineligible for FT analysis, such as those with artifacts or suboptimal presentation of ventricular contours. The present study was approved by the local institutional review board, and the requirement for subject consent was waived by the Chinese Ethics Committee (Unique identifier: ChiECRCT20190198).

### CMR image acquisition

CMR images were acquired with 1.5T CMR scanners (Signa HDxt, General Electric Healthcare, Chicago, Illinois, USA and Magnetom Essenza, Siemens Healthineers, Erlangen, Germany) using 16-channel phased-array surface coil. The subjects were placed in a supine position, and ECG gated balanced steady-state free precession (bSSFP) sequences were used to acquire cine images in held end-expiration. ECG-gated contiguous parallel short-axis planes (10–15 slices) were acquired throughout the LV and RV, covering the base (atrioventricular valve plane) to the apex. Long axis cine images of two-chamber, three-chamber, and four-chamber views were also acquired.

The parameters of the sequence for the Signa HDxt (General Electric Healthcare) were as follows: repetition time = 4 ms, echo time = 1.75 ms, flip angle = 60°, field of view = 310 × 310 mm, matrix size = 224 × 224, slice thickness = 8 mm, slice gap = 1 mm, and 30 phases per cardiac cycle. The scanning parameters for the Magnetom Essenza (Siemens Healthineers) were as follows: repetition time = 4.38 ms, echo time = 1.37 ms, flip angle = 60°, field of view = 275 × 340 mm, matrix size = 224 × 256, slice thickness = 8 mm, slice gap = 0 mm, and 30 phases per cardiac cycle.

### CMR-FT analysis

**Fig. 1:**
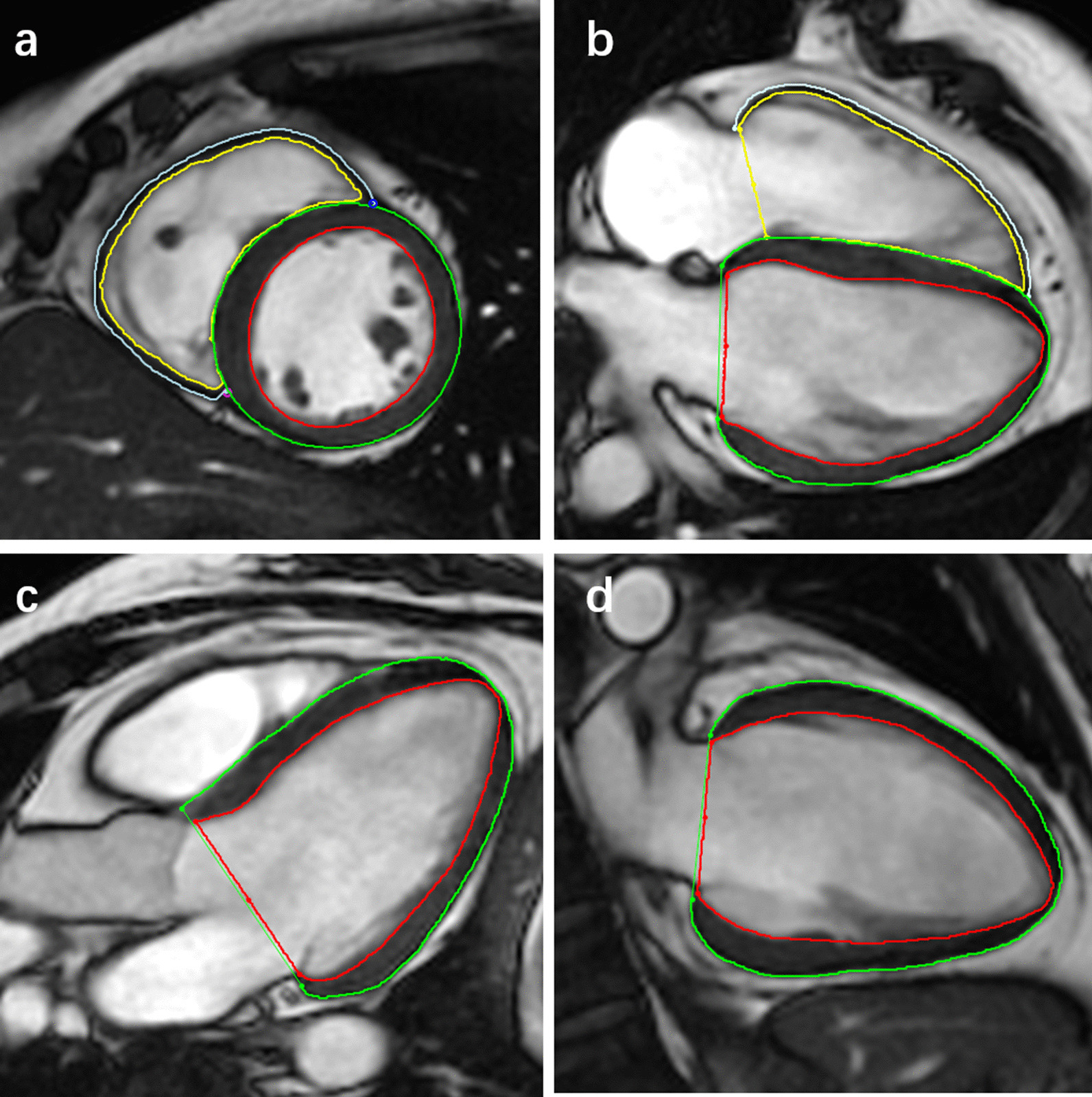
Example of biventricular myocardial deformation analysis by cvi^42^ (Circle Cardiovascular Imaging, Calgary, Canada). Contours are illustrated in left ventricular (LV) and right ventricular (RV) endocardial and epicardial borders in short-axis view (**a**), and four-chamber view (**b**), three-chamber view (**c**), and two-chamber view (**d**)

Image analysis was performed by an experienced researcher (GX. L, with more than 3 years of CMR experience using commercial software (cvi^42^^®^ version 5.12.1, Circle Cardiovascular Imaging, Calgary, Canada). The two-chamber, three-chamber, four-chamber, and short-axis cine images were uploaded to the software package. Datasets were anonymized to ensure that operators were blinded to all protected health information. The phase with the largest blood pool area in the middle of the LV or RV was defined as the end-diastolic phase in the short-axis images. The threshold-based segmentation method was used to depict the endocardium of the LV and RV, and then the contours of the epicardium of the LV and RV were manually drawn. The RV excluded the septum. The trabeculae and papillary muscles were included in the blood pool. The reference points were drawn manually at the insertion sites of the LV and RV superior and inferior septum for regional and global strain analysis and polar map generation. Next, guided by homogeneity or characteristic anatomical signals, the software automatically outlined the contour by tracking myocardial voxel points for the rest of the cardiac cycle. The performance of CMR-FT was visually reviewed to ensure accurate tracking. In the event of insufficient or erroneous tracking, the software allows boundary editing of the end-diastolic phase. Finally, the software automatically generated the biventricular global radial, circumferential, longitudinal strain (GRS, GCS, and GLS), peak systolic SR (PSSR) for the radial, circumferential, and longitudinal directions (PSSRR, PSSRC, PSSRL), and peak diastolic SR (PDSR) for the radial, circumferential, and longitudinal directions (PDSRR, PDSRC, PDSRL). In addition, radial and circumferential strain at the basal, mid-cavity, and apical levels of both the LV and RV were obtained (Fig. 1).

To explore the effect of analysis software on the measurements and further validate the associations of age and sex with biventricular strain values, we randomly selected 10 samples from each male and female age group (21–30, 31–40, 41–50, 51–60, and 61–70 years) using stratified sampling (100 cases in total), and analyzed the data using another widely recognized software (version 4.0.38.4, Medis Medical Imaging, Leiden, the Netherlands). Biventricular global strain metrics were assessed by the first author (GXL). The frames with the smallest blood pool in the middle of the LV or RV cavity were defined as the end-systolic phase and the largest as the end-diastolic phase. On the two-chamber, three-chamber, four-chamber, and short-axis cine imaging, LV endocardial borders, and the epicardial borders were manually traced using the point-and-click method. RV endocardial border was traced in the four-chamber view. Parameters of LV GRS, GCS, GLS and RV GLS of the free wall (RVFW-GLS) were obtained automatically (Additional file 1: Fig. S1).

### Reproducibility

Sixty subjects were randomly selected from the study populations, and interobserver reproducibility of biventricular strain metrics assessed by both CMR-FT software packages (cvi^42^, Circle Cardiovascular Imaging and Medis Medical Imaging) was evaluated by two independent experienced researchers (GXL and YYG, with more than 3 and 5 years of experience in CMR, respectively). To assess intraobserver reproducibility, the datasets were measured by a researcher (GXL) for the second time using the same method with a time interval of more than a month. In addition, we further examined the reproducibility of biventricular global strain measurements assessed by the manual contouring method used in the present study and the automatic contouring method provided by cvi^42^ software.

To assess the inter-scanner reproducibility of strain measurements, we re-recruited 20 healthy individuals to determine whether the obtained reference values were dependent on CMR scanner. Written informed consent was provided by all subjects. Each of the subjects underwent CMR examinations on the same day with the two CMR scanners using the same protocols as described. GRS, GCS, and GLS of both ventricles were measured by the first author (G.X. L) using cvi^42^ software (Circle Cardiovascular Imaging) based on the same method described above.

### Statistical analysis

All statistical analyses were performed with SPSS (version 26.0, Statistical Package for the Social Sciences, International Business Machines, Inc., Armonk, New York, USA) and GraphPad Prism (version 8.0, Graph-Pad Software, La Jolla, California, USA). The Shapiro–Wilk test was employed to assess the distribution of continuous variables. All normally distributed continuous variables are presented as the mean ± standard deviation (SD), and nonnormally distributed variables are expressed as the median (interquartile range). The enrolled subjects were stratified by sex (men and women) and 10-year age groups (21–30, 31–40, 41–50, 51–60, and 61–70 years). Sex differences in demographic characteristics and biventricular deformation parameters were analyzed using Student’s *t* test or the Mann–Whitney *U* test. Linear regression analysis was performed to assess the associations of biventricular global strain and SR with age. One-way analysis of variance (ANOVA) was performed to evaluate whether regional strain differed according to age groups. Differences in the basal, mid-cavity, and apical biventricular strain measurements were evaluated using ANOVA followed by Tukey’s post hoc test. Intra- and interobserver reproducibility was assessed by the intraclass correlation coefficient (ICC). A paired sample *t* test was used to examine the difference in strain parameters obtained by the two software packages (cvi^42^ Circle Cardioivascular Imaging and Medis Medical Imaging) and the two CMR scanners. Differences were regarded as statistically significant at *P* < 0.05.

## Results

### Baseline demographics

**Fig. 2:**
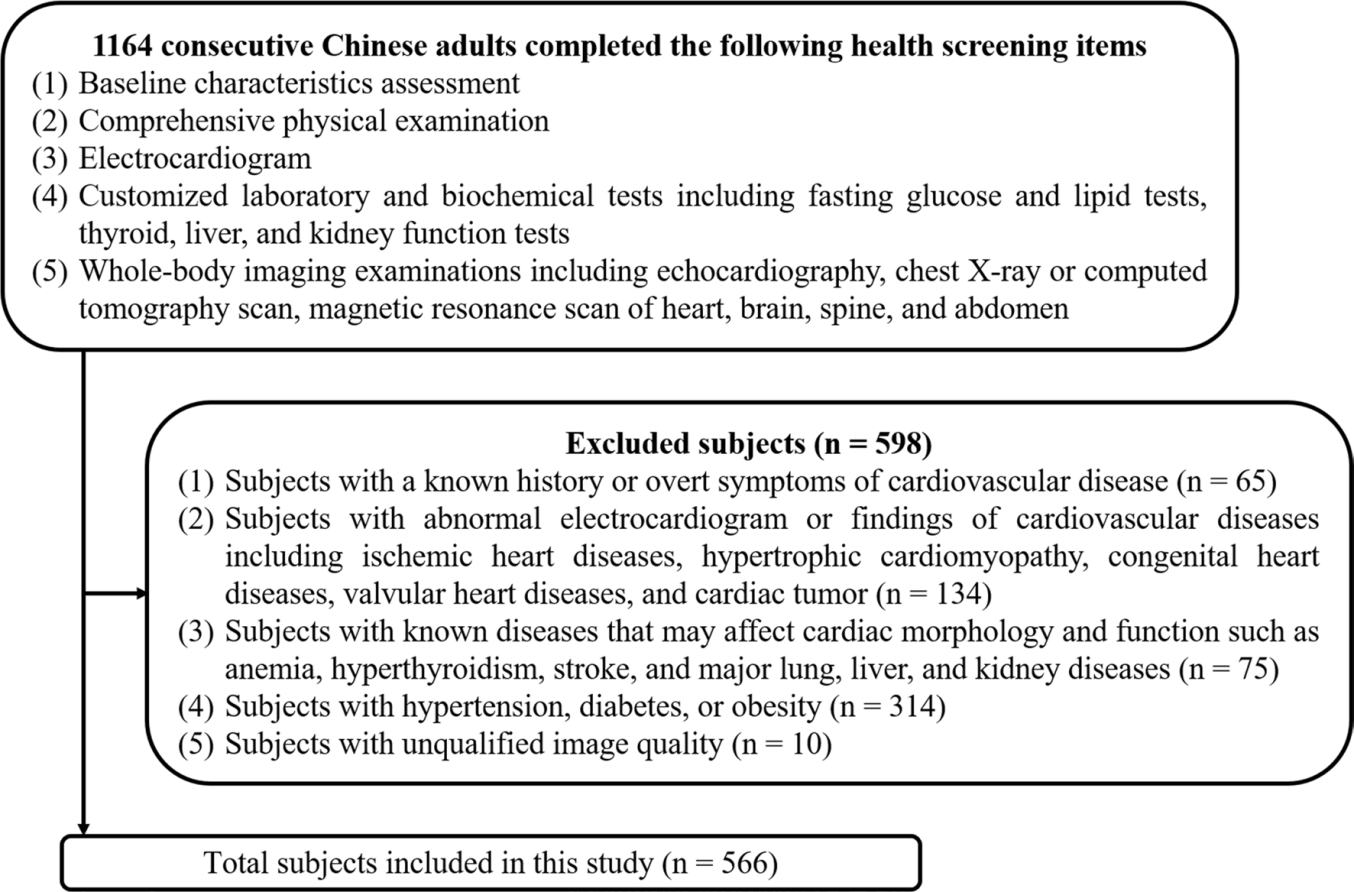
Case selection flowchart

A total of 1164 CMR examinations underwent image analysis. According to the exclusion criteria, 598 subjects were excluded, leaving 566 healthy adults (312 men) (Fig. 2). Of the 566 subjects, 306 subjects underwent CMR imaging with General Electric scanner and 260 subjects with Siemens Healthineers scanner. The demographic characteristics of the entire cohort are shown in Table 1. Men had higher height, BMI, body surface area (BSA), SBP, DBP, and serum levels of FBG, triglycerides, and low-density lipoprotein cholesterol than women (all *P* ≤ 0.001). Compared with men, women had higher high-density lipoprotein cholesterol levels and heart rate (both* P* ≤ 0.004). There was no significant sex difference in age or total cholesterol (*P* = 0.833 and *P* = 0.280, respectively).

**Table 1:** Demographic characteristics of the study population

Variables	All (n = 566)	Men (n = 312)	Women (n = 254)	*P* value (gender)
Age (years)	42.7 ± 10.7	42.7 ± 10.4	42.8 ± 11.1	0.833
Height (cm)	166.3 ± 8.3	171.5 ± 6.1	160.0 ± 6.0	< 0.001
BMI (kg/m^2^)	22.9 ± 3.0	23.7 ± 2.6	21.9 ± 3.0	< 0.001
BSA (m^2^)	1.71 ± 0.18	1.83 ± 0.14	1.58 ± 0.12	< 0.001
HR (bpm)	67 ± 9	66 ± 9	68 ± 9	0.004
SBP (mm Hg)	114 ± 13	117 ± 11	109 ± 14	< 0.001
DBP (mm Hg)	70.5 ± 9.5	72.9 ± 8.6	67.4 ± 9.6	< 0.001
FBG (mmol/L)	5.19 ± 0.46	5.25 ± 0.45	5.13 ± 0.46	0.001
TC (mmol/L)	5.00 ± 0.91	5.04 ± 0.94	4.96 ± 0.88	0.280
TG (mmol/L)	1.13 (0.81–1.65)	1.36 (0.97–1.94)	0.91 (0.73–1.25)	< 0.001
HDL-C (mmol/L)	1.37 (1.17–1.65)	1.25 (1.06–1.45)	1.56 (1.32–1.90)	< 0.001
LDL-C (mmol/L)	2.84 ± 0.90	2.96 ± 0.91	2.69 ± 0.84	< 0.001

Normally distributed continuous variables are presented as mean ± standard deviation (SD) and non-normally distributed variables are expressed as median (interquartile range). *P* value is for Student’s *t*-test between genders

BMI, body mass index; BSA, body surface area; HR, heart rate; SBP, systolic blood pressure DBP, diastolic blood pressure; FBG, fasting blood glucose; TC, total cholesterol; TG, triglycerides; HDL-C, high-density lipoprotein cholesterol; LDL-C, low-density lipoprotein cholesterol

### Sex difference in biventricular deformation parameters

LV and RV global strain and the SRs for men and women assessed by cvi^42^ are shown in Table 2. For the LV, women showed greater magnitudes of GRS (37.6 ± 6.1% vs. 32.1 ± 5.3%), GCS (− 20.7 ± 1.9% vs. − 18.8 ± 1.9%), and GLS (− 17.8 ± 1.8% vs. − 15.6 ± 1.8%) (all *P* < 0.001). Similarly, for the RV, women had greater magnitudes of GRS (25.1 ± 7.8% vs. 22.1 ± 6.7%), GCS (− 14.4 ± 3.6% vs. − 13.2 ± 3.2%), and GLS (− 22.4 ± 5.2% vs. − 20.2 ± 4.6%) (all *P* < 0.001). The magnitudes of biventricular PSSR in women were all significantly (all *P* < 0.05). Specifically, the LV PSSR for men and women was 2.20 ± 0.67 s^−1^ vs. 2.56 ± 0.75 s^−1^ for the radial direction, − 1.12 ± 0.22 s^−1^ vs. − 1.25 ± 0.25 s^−1^ for the circumferential direction, and − 0.90 ± 0.17 s^−1^ vs. − 1.03 ± 0.19 s^−1^ for the longitudinal direction. The RV PSSR for men and women was 1.46 ± 0.64 s^−1^ vs. 1.60 ± 0.73 s^−1^ for the radial direction, − 0.78 ± 0.26 s^−1^ vs. − 0.82 ± 0.28 s^−1^ for the circumferential direction, and − 1.16 ± 0.33 s^−1^ vs. − 1.31 ± 0.30 s^−1^ for the longitudinal direction, respectively. In addition, the magnitudes of biventricular PDSR in women were all greater (all *P* < 0.001). The LV PDSR for men and women was − 1.77 ± 0.40 s^−1^ vs. − 2.23 ± 0.50 s^−1^ for the radial direction, 0.94 ± 0.18 s^−1^ vs. 1.09 ± 0.21 s^−1^ for the circumferential direction, and 0.75 ± 0.16 s^−1^ vs. 0.91 ± 0.19 s^−1^ for the longitudinal direction, respectively. The RV PDSR between men and women was − 1.01 ± 0.33 s^−1^ vs. − 1.30 ± 0.45 s^−1^ for the radial direction, 0.58 ± 0.18 s^−1^ vs. 0.67 ± 0.19 s^−1^ for the circumferential direction, and 0.94 ± 0.27 s^−1^ vs. 1.06 ± 0.33 s^−1^ for the longitudinal direction, respectively. The measurements for biventricular regional strain for both sexes are presented in Table 3. The absolute values of basal, mid-cavity and apical biventricular strain in women were all significantly greater (all *P* ≤ 0.001). In general, the magnitudes of biventricular circumferential and radial strain decreased from apex to base (all *P* < 0.01).

**Table 2:** Normal values of biventricular global strain and strain rates for men and women

Variables	All (n = 566)	Men (n = 312)	Women (n = 254)	*P* value (gender)
Left ventricle				
GRS (%)	34.5 ± 6.3	32.1 ± 5.3	37.6 ± 6.1	< 0.001
GCS (%)	− 19.6 ± 2.1	− 18.8 ± 1.9	− 20.7 ± 1.9	< 0.001
GLS (%)	− 16.6 ± 2.1	− 15.6 ± 1.8	− 17.8 ± 1.8	< 0.001
PSSRR (s^−1^)	2.37 ± 0.73	2.20 ± 0.67	2.56 ± 0.75	< 0.001
PSSRC (s^−1^)	− 1.18 ± 0.24	− 1.12 ± 0.22	− 1.25 ± 0.25	< 0.001
PSSRL (s^−1^)	− 0.96 ± 0.19	− 0.90 ± 0.17	− 1.03 ± 0.19	< 0.001
PDSRR (s^−1^)	− 1.98 ± 0.50	− 1.77 ± 0.40	− 2.23 ± 0.50	< 0.001
PDSRC (s^−1^)	1.01 ± 0.21	0.94 ± 0.18	1.09 ± 0.21	< 0.001
PDSRL (s^−1^)	0.82 ± 0.19	0.75 ± 0.16	0.91 ± 0.19	< 0.001
Right ventricle				
GRS (%)	23.4 ± 7.4	22.1 ± 6.7	25.1 ± 7.8	< 0.001
GCS (%)	− 13.8 ± 3.4	− 13.2 ± 3.2	− 14.4 ± 3.6	< 0.001
GLS (%)	− 21.2 ± 5.0	− 20.2 ± 4.6	− 22.4 ± 5.2	< 0.001
PSSRR (s^−1^)	1.52 ± 0.68	1.46 ± 0.64	1.60 ± 0.73	0.012
PSSRC (s^−1^)	− 0.80 ± 0.27	− 0.78 ± 0.26	− 0.82 ± 0.28	0.042
PSSRL (s^−1^)	− 1.23 ± 0.33	− 1.16 ± 0.33	− 1.31 ± 0.30	< 0.001
PDSRR (s^−1^)	− 1.14 ± 0.42	− 1.01 ± 0.33	− 1.30 ± 0.45	< 0.001
PDSRC (s^−1^)	0.62 ± 0.19	0.58 ± 0.18	0.67 ± 0.19	< 0.001
PDSRL (s^−1^)	0.99 ± 0.30	0.94 ± 0.27	1.06 ± 0.33	< 0.001

Measurements of biventricular global strain and strain rates were assessed by cvi^42^. Data are presented as means ± standard deviation. *P* value is for Student’s t-test between genders

GRS, global peak radial strain; GCS, global peak circumferential strain; GLS, global peak longitudinal strain; PSSRR, peak systolic strain rate radial; PSSRC, peak systolic strain rate circumferential; PSSRL, peak systolic strain rate longitudinal; PDSRR, peak diastolic strain rate radial; PDSRC, peak diastolic strain rate circumferential; PDSRL, peak diastolic strain rate longitudinal

**Table 3:** Normal values of biventricular regional strain for men and women

Variables	All (n = 566)	Men (n = 312)	Women (n = 254)	*P* value (gender)
LV radial strain (%)				
Basal	32.1 ± 6.1	29.5 ± 5.0	35.4 ± 5.6	< 0.001
Mid-cavity	32.9 ± 6.7	30.8 ± 5.7*	35.4 ± 6.9	< 0.001
Apical	50.6 ± 11.1*^†^	48.2 ± 10.6*^†^	53.6 ± 11.1*^†^	< 0.001
LV circumferential strain (%)				
Basal	− 18.7 ± 2.3	− 17.8 ± 2.1	− 19.9 ± 1.9	< 0.001
Mid-cavity	− 19.3 ± 2.4*	− 18.6 ± 2.2*	− 20.2 ± 2.4	< 0.001
Apical	− 24.1 ± 2.8*^†^	− 23.5 ± 2.7*^†^	− 24.9 ± 2.6*^†^	< 0.001
RV radial strain (%)				
Basal	18.7 ± 6.4	17.5 ± 5.9	20.2 ± 6.8	< 0.001
Mid-cavity	26.0 ± 9.0*	24.4 ± 8.1*	27.8 ± 9.7*	< 0.001
Apical	35.3 ± 13.1*^†^	33.7 ± 12.2*^†^	37.3 ± 13.9*^†^	0.001
RV circumferential strain (%)				
Basal	− 11.1 ± 3.5	− 10.6 ± 3.3	− 11.8 ± 3.5	< 0.001
Mid-cavity	− 15.4 ± 4.0*	− 14.9 ± 3.8*	− 16.0 ± 4.1*	0.001
Apical	− 18.6 ± 4.9*^†^	− 18.0 ± 4.9*^†^	− 19.4 ± 4.8*^†^	0.001

Measurements of biventricular regional strain were assessed by cvi^42^. Data are presented as means ± standard deviation. *P* value is for Student’s t-test between genders. One-way analysis of variance is used to test the differences between basal, mid-cavity, and apical biventricular strain measurements

LV, left ventricular; RV, right ventricular

^*^P < 0.01 vs. basal level. ^†^*P* < 0.01 vs. mid-cavity level

The sex difference in biventricular global strain assessed by Medis are presented in Additional file 3: Table S1. Specifically, except for LV GRS (*P* = 0.808), the magnitudes of all strain metrics, including LV GCS, GLS, and RVFW-GLS, were greater in women (all *P* < 0.001), which was generally consistent with the results measured by cvi^42^.

### Correlation between age and biventricular deformation parameters

**Fig. 3:**
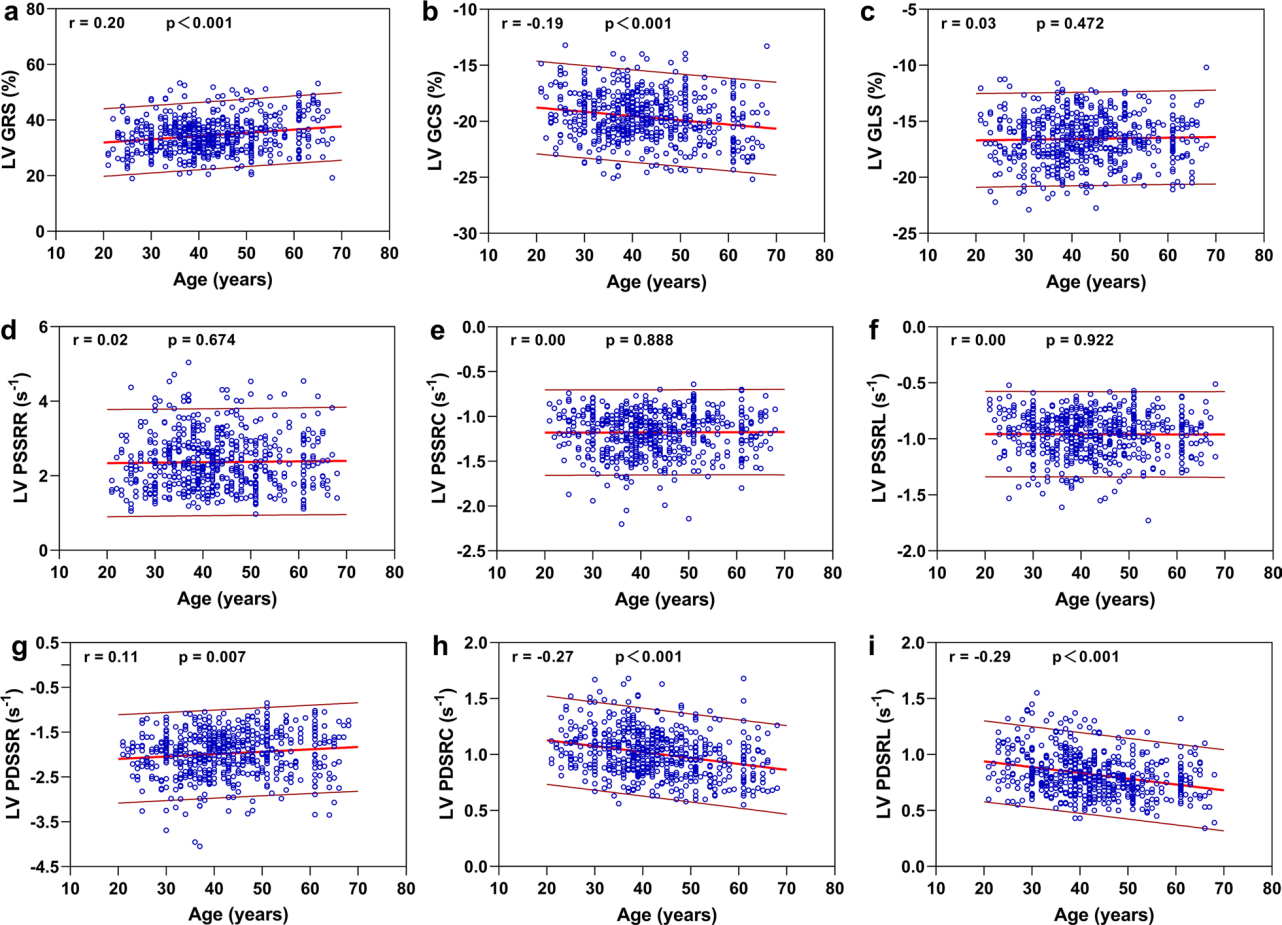
Correlation between left ventricular global strain and systolic, diastolic strain rate and age. Linear regressions with 95% prediction band for LV global radial strain (GRS) (**a**), global circumferential strain (GCS) (**b**), global longitudinal strain (GLS) (**c**), peak systolic strain rate radial (PSSRR) (**d**), peak systolic strain rate circumferential (PSSRC) (**e**), peak systolic strain rate longitudinal (PSSRL) (**f**), peak diastolic strain rate radial (PDSRR) (**g**), peak diastolic strain rate circumferential (PDSRC) (**h**), and peak diastolic strain rate longitudinal (PDSRL) (**i**)

**Fig. 4:**
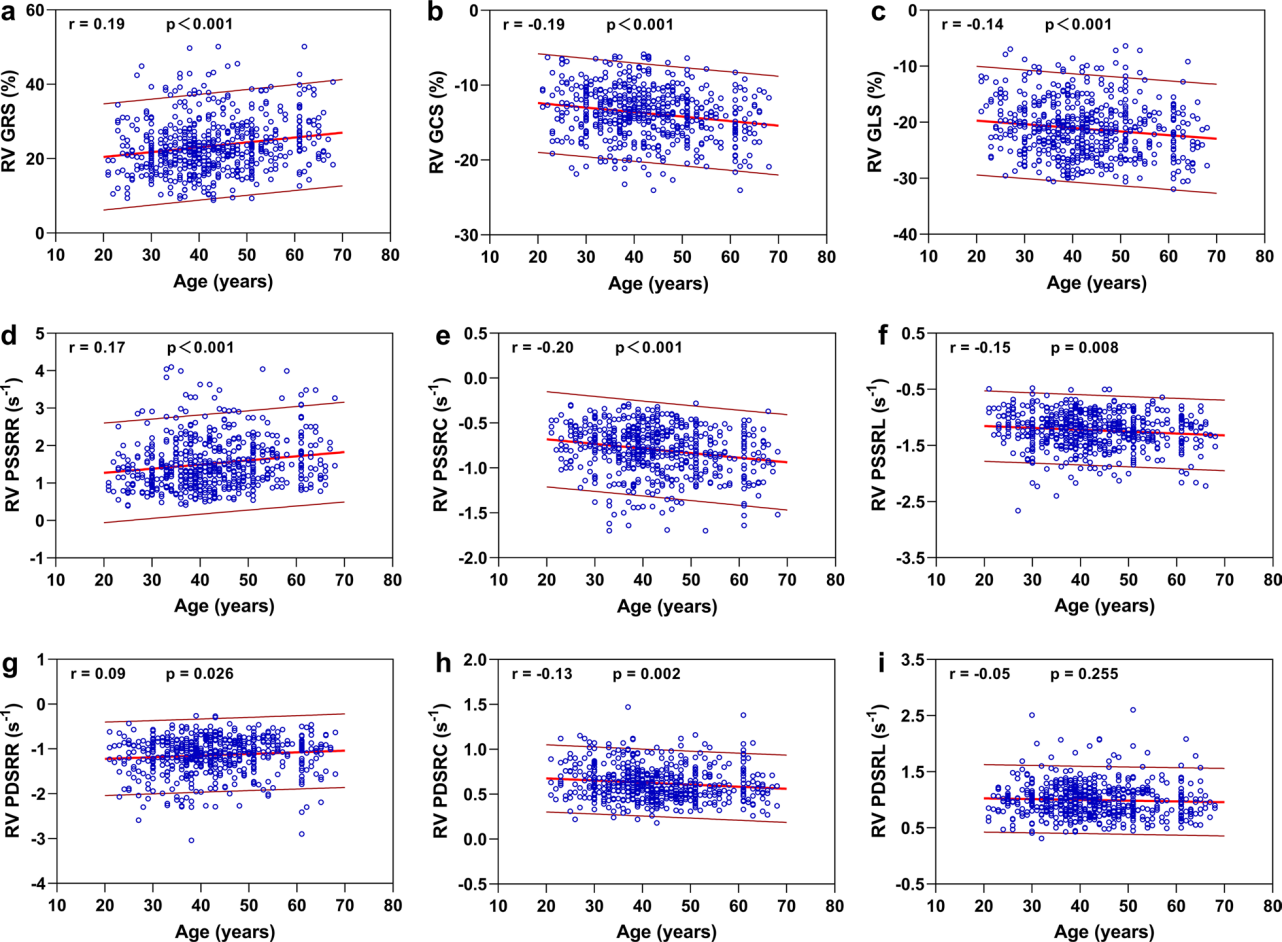
Correlation between right ventricular global strain and systolic, diastolic strain rate and age. Linear regressions with 95% prediction band for RV global radial strain (GRS) (**a**), global circumferential strain (GCS) (**b**), global longitudinal strain (GLS) (**c**), peak systolic strain rate radial (PSSRR) (**d**), peak systolic strain rate circumferential (PSSRC) (**e**), peak systolic strain rate longitudinal (PSSRL) (**f**), peak diastolic strain rate radial (PDSRR) (**g**), peak diastolic strain rate circumferential (PDSRC) (**h**), and peak diastolic strain rate longitudinal (PDSRL) (**i**)

The sex-specific normal values for global myocardial strain and SR according to age decades measured by cvi^42^ are shown in Tables 4 and 5. For the LV, with advancing age, there was a linear increase in the magnitudes of GRS and GCS, whereas the magnitudes of PDSRR, PDSRC, and PDSRL showed a decrease with age (all *P* < 0.01). There was no significant association of GLS or PSSR with age (Fig. 3). Regarding the RV, aging was associated with an increase in the magnitudes of GRS, GCS, GLS, PSSRR, PSSRC, and PSRSRL and a decrease in the magnitudes of PDSRR and PDSRC (all *P* < 0.05). Age showed no significant correlation with PDSRL (Fig. 4). The sex-specific measurements of biventricular regional strain by 10-year age groups are shown in Tables 6 and 7. All the measurements of biventricular strain at the basal, mid-cavity, and apical levels were significantly associated with age in both sexes (all *P* < 0.05).

**Table 4:** Normal values of biventricular global strain and strain rates by age decades for men

Variables	21–30 years (n = 44)	31–40 years (n = 100)	41–50 years (n = 92)	51–60 years (n = 48)	61–70 years (n = 28)
Left ventricle					
GRS (%)	29.8 ± 4.7	32.1 ± 5.0	32.6 ± 5.1	31.8 ± 5.1	34.6 ± 7.4
GCS (%)	− 17.9 ± 1.8	− 18.8 ± 1.8	− 19.0 ± 1.8	− 18.8 ± 1.9	− 19.6 ± 2.5
GLS (%)	− 15.3 ± 1.8	− 15.8 ± 1.8	− 15.5 ± 1.7	− 15.4 ± 1.9	− 15.6 ± 2.0
PSSRR (s^−1^)	1.97 ± 0.56	2.31 ± 0.69	2.27 ± 0.68	2.02 ± 0.61	2.31 ± 0.76
PSSRC (s^−1^)	− 1.04 ± 0.18	− 1.15 ± 0.25	− 1.15 ± 0.25	− 1.06 ± 0.21	− 1.13 ± 0.22
PSSRL (s^−1^)	− 0.87 ± 0.22	− 0.92 ± 0.17	− 0.91 ± 0.17	− 0.88 ± 0.17	− 0.91 ± 0.16
PDSRR (s^−1^)	− 1.80 ± 0.35	− 1.81 ± 0.39	− 1.77 ± 0.35	− 1.64 ± 0.40	− 1.75 ± 0.59
PDSRC (s^−1^)	0.99 ± 0.14	0.97 ± 0.18	0.96 ± 0.18	0.87 ± 0.15	0.84 ± 0.20
PDSRL (s^−1^)	0.81 ± 0.15	0.78 ± 0.16	0.74 ± 0.15	0.69 ± 0.15	0.67 ± 0.14
Right ventricle					
GRS (%)	20.1 ± 5.9	21.6 ± 6.7	22.0 ± 6.7	23.5 ± 6.5	24.6 ± 8.0
GCS (%)	− 12.4 ± 3.1	− 13.0 ± 3.1	− 13.2 ± 3.2	− 14.0 ± 2.9	− 14.2 ± 3.5
GLS (%)	− 18.2 ± 5.0	− 20.1 ± 4.7	− 21.1 ± 4.0	− 20.2 ± 4.5	− 20.9 ± 4.6
PSSRR (s^−1^)	1.23 ± 0.45	1.43 ± 0.69	1.49 ± 0.63	1.57 ± 0.60	1.64 ± 0.74
PSSRC (s^−1^)	− 0.70 ± 0.21	− 0.76 ± 0.27	− 0.78 ± 0.29	− 0.84 ± 0.24	− 0.85 ± 0.26
PSSRL (s^−1^)	− 1.06 ± 0.38	− 1.13 ± 0.36	− 1.19 ± 0.30	− 1.21 ± 0.26	− 1.25 ± 0.37
PDSRR (s^−1^)	− 1.07 ± 0.39	− 1.04 ± 0.34	− 0.99 ± 0.27	− 0.94 ± 0.27	− 0.96 ± 0.41
PDSRC (s^−1^)	0.62 ± 0.19	0.60 ± 0.19	0.55 ± 0.15	0.57 ± 0.18	0.57 ± 0.21
PDSRL (s^−1^)	0.91 ± 0.25	0.94 ± 0.25	0.96 ± 0.28	0.91 ± 0.26	0.97 ± 0.33

Measurements of biventricular global strain and strain rates were assessed by cvi^42^. Data are presented as means ± standard deviation

GRS, global peak radial strain; GCS, global peak circumferential strain; GLS, global peak longitudinal strain; PSSRR, peak systolic strain rate radial; PSSRC, peak systolic strain rate circumferential; PSSRL, peak systolic strain rate longitudinal; PDSRR, peak diastolic strain rate radial; PDSRC, peak diastolic strain rate circumferential; PDSRL, peak diastolic strain rate longitudinal

**Table 5:** Normal values of biventricular global strain and strain rates by age decades for women

Variables	21–30 years (n = 40)	31–40 years(n = 78)	41–50 years (n = 71)	51–60 years (n = 34)	61–70 years (n = 31)
Left ventricle					
GRS (%)	35.5 ± 5.4	37.3 ± 5.7	37.0 ± 6.3	38.3 ± 5.8	41.2 ± 6.2
GCS (%)	− 20.0 ± 1.8	− 20.6 ± 1.8	− 20.5 ± 2.0	− 20.9 ± 1.8	− 21.7 ± 1.9
GLS (%)	− 18.0 ± 2.0	− 18.2 ± 1.8	− 17.6 ± 1.9	− 17.2 ± 1.5	− 17.8 ± 1.8
PSSRR (s^−1^)	2.40 ± 0.78	2.67 ± 0.78	2.61 ± 0.71	2.51 ± 0.68	2.43 ± 0.77
PSSRC (s^−1^)	− 1.19 ± 0.26	− 1.32 ± 0.27	− 1.25 ± 0.21	− 1.22 ± 0.22	− 1.19 ± 0.25
PSSRL (s^−1^)	− 1.00 ± 0.19	− 1.05 ± 0.20	− 1.05 ± 0.18	− 0.99 ± 0.21	− 1.01 ± 0.17
PDSRR (s^−1^)	− 2.28 ± 0.47	− 2.37 ± 0.50	− 2.15 ± 0.49	− 2.18 ± 0.44	− 2.08 ± 0.55
PDSRC (s^−1^)	1.18 ± 0.21	1.15 ± 0.20	1.04 ± 0.18	1.02 ± 0.20	0.99 ± 0.22
PDSRL (s^−1^)	1.05 ± 0.20	0.97 ± 0.18	0.84 ± 0.17	0.82 ± 0.17	0.85 ± 0.15
Right ventricle					
GRS (%)	24.1 ± 7.7	24.3 ± 7.6	24.2 ± 7.6	25.7 ± 8.4	29.6 ± 6.6
GCS (%)	− 14.2 ± 3.3	− 13.9 ± 3.6	− 14.0 ± 3.4	− 14.0 ± 3.8	− 16.4 ± 2.9
GLS (%)	− 21.7 ± 4.4	− 22.5 ± 5.1	− 21.8 ± 5.6	− 22.4 ± 5.5	− 24.3 ± 4.6
PSSRR (s^−1^)	1.40 ± 0.64	1.57 ± 0.77	1.58 ± 0.70	1.75 ± 0.72	1.85 ± 0.74
PSSRC (s^−1^)	− 0.72 ± 0.25	− 0.81 ± 0.30	− 0.81 ± 0.26	− 0.88 ± 0.25	− 0.96 ± 0.31
PSSRL (s^−1^)	− 1.30 ± 0.29	− 1.29 ± 0.31	− 1.32 ± 0.31	− 1.27 ± 0.31	− 1.37 ± 0.30
PDSRR (s^−1^)	− 1.41 ± 0.42	− 1.37 ± 0.46	− 1.20 ± 0.44	− 1.20 ± 0.44	− 1.35 ± 0.49
PDSRC (s^−1^)	0.75 ± 0.20	0.70 ± 0.18	0.62 ± 0.18	0.65 ± 0.22	0.65 ± 0.22
PDSRL (s^−1^)	1.12 ± 0.33	1.11 ± 0.32	1.03 ± 0.31	1.06 ± 0.43	0.96 ± 0.29

Measurements of biventricular global strain and strain rates were assessed by cvi^42^. Data are presented as means ± standard deviation

GRS, global peak radial strain; GCS, global peak circumferential strain; GLS, global peak longitudinal strain; PSSRR, peak systolic strain rate radial; PSSRC, peak systolic strain rate circumferential; PSSRL, peak systolic strain rate longitudinal; PDSRR, peak diastolic strain rate radial; PDSRC, peak diastolic strain rate circumferential; PDSRL, peak diastolic strain rate longitudinal

**Table 6:** Normal values of biventricular regional strain by age decades for men

Variables	21–30 years (n = 44)	31–40 years (n = 100)	41–50 years (n = 92)	51–60 years (n = 48)	61–70 years (n = 28)	*P* value
LV radial strain (%)						
Basal	26.2 ± 5.1	29.4 ± 4.4	30.0 ± 4.3	29.4 ± 5.5	33.3 ± 5.5	< 0.001
Mid-cavity	28.5 ± 5.3	30.9 ± 5.5	30.9 ± 5.2	30.5 ± 5.2	34.7 ± 7.8	< 0.001
Apical	45.4 ± 9.7	48.1 ± 10.8	50.4 ± 10.5	45.7 ± 9.6	49.3 ± 12.3	0.043
LV circumferential strain (%)						
Basal	− 16.3 ± 2.4	− 17.8 ± 1.7	− 18.0 ± 1.7	− 17.7 ± 2.2	− 19.4 ± 2.3	< 0.001
Mid-cavity	− 17.6 ± 2.2	− 18.5 ± 2.1	− 18.6 ± 1.9	− 18.4 ± 1.9	− 20.4 ± 2.9	< 0.001
Apical	− 22.6 ± 2.9	− 23.4 ± 2.7	− 24.1 ± 2.7	− 23.0 ± 2.5	− 24.4 ± 2.9	0.009
RV radial strain (%)						
Basal	15.5 ± 5.9	17.0 ± 5.6	17.4 ± 5.6	18.4 ± 5.8	21.0 ± 6.3	0.002
Mid-cavity	22.1 ± 7.5	23.6 ± 7.7	24.1 ± 8.4	26.1 ± 7.6	29.6 ± 8.4	0.001
Apical	30.8 ± 9.4	33.2 ± 12.6	32.7 ± 12.7	36.0 ± 12.3	39.2 ± 10.9	0.030
RV circumferential strain (%)						
Basal	− 9.6 ± 3.3	− 10.1 ± 3.1	− 10.4 ± 3.0	− 11.1 ± 3.0	− 13.6 ± 4.1	< 0.001
Mid-cavity	− 13.9 ± 3.6	− 14.5 ± 3.6	− 14.7 ± 3.8	− 15.6 ± 3.2	− 18.1 ± 4.0	< 0.001
Apical	− 16.9 ± 5.8	− 17.8 ± 4.5	− 17.5 ± 5.2	− 18.7 ± 4.1	− 21.2 ± 4.0	0.002

Measurements of biventricular regional strain were assessed by cvi^42^

Data are presented as means ± standard deviation. *P* value are from analysis of variance test with regression

LV, left ventricular; RV, right ventricular

**Table 7:** Normal values of biventricular regional strain by age decades for women

Variables	21–30 years (n = 40)	31–40 years (n = 78)	41–50 years (n = 71)	51–60 years (n = 34)	61–70 years (n = 31)	*P* value
LV radial strain (%)						
Basal	33.4 ± 5.4	34.6 ± 5.3	35.2 ± 5.5	37.3 ± 5.4	38.8 ± 5.4	< 0.001
Mid-cavity	33.0 ± 5.8	34.6 ± 6.1	34.7 ± 7.5	36.1 ± 6.3	41.8 ± 5.6	< 0.001
Apical	51.0 ± 13.0	54.0 ± 8.6	53.0 ± 12.5	52.2 ± 9.7	58.9 ± 10.0	0.036
LV circumferential strain (%)						
Basal	− 19.2 ± 2.1	− 19.7 ± 1.9	− 19.8 ± 1.9	− 20.5 ± 1.6	− 21.0 ± 1.7	< 0.001
Mid-cavity	− 19.3 ± 2.1	− 19.9 ± 2.0	− 19.8 ± 2.7	− 20.4 ± 2.1	− 22.6 ± 1.7	< 0.001
Apical	− 24.2 ± 3.1	− 25.2 ± 2.2	− 24.6 ± 2.9	− 24.5 ± 2.2	− 25.9 ± 2.6	0.028
RV radial strain (%)						
Basal	20.9 ± 7.0	20.1 ± 6.2	18.6 ± 7.2	19.9 ± 6.8	23.6 ± 5.7	0.013
Mid-cavity	25.7 ± 8.6	25.8 ± 9.2	27.0 ± 10.0	31.0 ± 8.7	34.0 ± 9.7	< 0.001
Apical	33.9 ± 15.1	36.8 ± 14.8	36.7 ± 13.2	37.9 ± 11.9	44.0 ± 11.8	0.040
RV circumferential strain (%)						
Basal	− 12.0 ± 3.5	− 11.8 ± 3.1	− 11.0 ± 3.8	− 11.7 ± 3.9	− 13.8 ± 2.8	0.006
Mid-cavity	− 15.2 ± 3.9	− 15.2 ± 3.9	− 15.7 ± 4.2	− 17.6 ± 3.7	− 18.4 ± 3.8	< 0.001
Apical	− 18.2 ± 5.0	− 19.2 ± 5.1	− 19.0 ± 4.8	− 19.6 ± 4.2	− 22.1 ± 3.7	0.011

Measurements of biventricular regional strain were assessed by cvi^42^

Data are presented as means ± standard deviation. *P* value from analysis of variance test with regression

LV, left ventricular; RV, right ventricular

The association of biventricular strain with age assessed by Medis was in line with that assessed by cvi^42^, confirming that aging was related to greater magnitudes of LV GRS, GCS, and RVFW-GLS (all *P* < 0.01), but had no significant correlation with LV GLS (*P* = 0.859) (Additional file 2: Fig. S2). The sex-specific biventricular global strain parameters stratified by 10-year age groups are presented in Additional file 3: Tables S2, S3.

### Effects of analysis software and CMR scanner vendor on strain measurements

A paired sample *t* test showed that compared with those assessed by cvi^42^, the magnitudes of LV global strain evaluated by Medis were significantly larger (all *P* < 0.01), especially for GRS and GLS. Specifically, LV GRS, GCS, and GLS evaluated by the two software packages were 35.5 ± 7.0% vs. 51.4 ± 17.3%, − 19.9 ± 2.3% vs. − 22.2 ± 3.4%, and − 16.8 ± 2.2% vs. − 22.6 ± 3.7%, respectively (Additional file 3: Table S4). In addition, we confirmed that there was no significant difference in the strain measurements derived from the two CMR scanners using a paired sample *t* test (all *P* > 0.05) (Additional file 3: Table S5).

### Reproducibility

The intra- and interobserver reproducibility values of the strain metrics assessed by cvi^42^ (Circle Cardiovascular Imaging) are shown in Table 8 and the reproducibility of biventricular deformation parameters obtained by manual and automatic contouring methods are shown in Additional file 3: Table S6. All the measurements of biventricular strain showed good to excellent reproducibility, with ICCs ranging from 0.86 to 0.97. In general, the strain measurements for the LV demonstrated slightly higher intra- and interobserver reproducibility than those for the RV. In addition, biventricular strain measurements, including LV GRS, GCS, GLS, and RVFW-GLS assessed by Medis, had acceptable intra- and interobserver reproducibility, with ICCs ranging from 0.79 to 0.97 (Additional file 3: Table S7). We further confirmed that measurements of biventricular GRS, GCS, and GLS obtained by the two CMR scanners were of good to excellent reproducibility, with ICCs ranging from 0.82 to 0.92 (Additional file 3: Table S8).

**Table 8:** Intra- and inter-observer reproducibility of LV and RV global strain parameters

Variables	Variability	Mean absolute bias	Limits of agreement	ICC (95% CI)
LV GRS (%)	Intraobserver	1.2 ± 1.5	− 2.6 to 4.7	0.97 (0.94, 0.98)
	Interobserver	1.6 ± 1.7	− 2.0 to 5.8	0.95 (0.92, 0.97)
LV GCS (%)	Intraobserver	− 0.4 ± 0.5	− 1.5 to 0.8	0.97 (0.95, 0.98)
	Interobserver	− 0.5 ± 0.4	− 1.8 to 0.7	0.96 (0.93, 0.98)
LV GLS (%)	Intraobserver	0.1 ± 0.9	− 2.7 to 3.4	0.91 (0.85, 0.94)
	Interobserver	− 0.3 ± 1.0	− 3.3 to 2.3	0.89 (0.83, 0.94)
RV GRS (%)	Intraobserver	0.4 ± 1.9	− 5.6 to 8.0	0.96 (0.94, 0.98)
	Interobserver	1.1 ± 2.3	− 6.6 to 8.1	0.95 (0.92, 0.97)
RV GCS (%)	Intraobserver	− 0.1 ± 1.2	− 3.4 to 3.5	0.94 (0.90, 0.96)
	Interobserver	− 0.6 ± 1.4	− 4.6 to 3.6	0.93 (0.88, 0.95)
RV GLS (%)	Intraobserver	0.3 ± 2.3	− 8.5 to 4.6	0.88 (0.81, 0.93)
	Interobserver	− 0.7 ± 2.7	− 5.8 to 6.7	0.86 (0.77, 0.91)

Measurements of biventricular global strain were assessed by cvi^42^

LV, left ventricular; RV, right ventricular; GRS, global peak radial strain; GLS, global peak longitudinal strain; GCS, global peak circumferential strain; SD, standard deviation; ICC, intra-class correlation coefficient; CI, confidence interval

## Discussion

Several CMR techniques, including tissue tagging, phase velocity mapping, displacement encoding with stimulated echoes (DENSE), and strain encoding (SENC), have been developed to assess myocardial deformation. However, all these methods have certain limitations, such as the need for additional acquisitions, low signal-to-noise ratio, and time-consuming postprocessing. CMR-FT, a well-established and continuously evolving technique based on conventional cine sequences, enables comprehensive cardiac performance quantification of all cardiac chambers, including the relatively thin-walled RV. In this CMR-FT study, we systematically obtained age- and sex-specific normal ranges of biventricular deformation parameters for all three linear components, derived from a large cohort of validated healthy Chinese adults across a broad age spectrum. In addition, we further confirmed that myocardial deformation metrics are influenced by both age and sex, as there were significant differences in the analyzed strata. Our findings may be used as a reference standard for the early detection of cardiovascular dysfunction using CMR-FT to inform the diagnosis, risk stratification, and therapeutic effect monitoring of cardiovascular diseases.

Although several studies have contributed to establishing normal reference values of biventricular strain and SR for Chinese populations, this study presents several advantages. First, as one of the most important factors affecting the validity of normal reference ranges, the sample size of this study is significantly larger than those of similar previous studies, e.g., Peng et al. [17] (n = 150), Liu et al. [18] (n = 120), and Qu et al. [19] (n = 150), and the larger sample size may strengthen the associations of myocardial deformation measurements with common demographic factors such as age and sex. Second, our selection criteria for “healthy” samples were more stringent than those used in previous studies. Specifically, all the enrolled subjects were asymptomatic and had no known cardiovascular disease or common comorbidities that may affect myocardial function, including hypertension, diabetes, and obesity. In addition, the included subjects were further confirmed to be healthy by a dedicated health screening visit, including comprehensive physical examinations, ECG, laboratory and biochemical examinations, and whole-body imaging examinations. Thus, the obtained normal reference values in this study should be more reliable. Finally, we included more comprehensive myocardial deformation metrics than previous studies, as biventricular strain and systolic and diastolic SR were systematically provided and further stratified by both sex and 10-year age groups. Currently, there is a growing body of literature showing that in some clinical scenarios, regional assessment of myocardial deformation may provide additional prognostic information over global measurements alone [25, 26]. In addition to global deformation parameters, we presented normal values of sex- and age-related regional biventricular radial and circumferential strain and confirmed that the magnitudes of these strain indices showed an overall increasing pattern from base to apex in both sexes. Therefore, this study can be used as an effective supplement to previous studies, thereby further promoting the extensive application of the CMR-FT technique in clinical practice and research.

### Correlation between sex and biventricular strain and SR

Multiple studies have explored the associations of biventricular deformation parameters with sex. For the LV, most studies confirmed that women have larger magnitudes of GLS and GCS [17, 27, 28], which was consistent with our findings. However, there are discrepant data regarding the relationship between GRS and sex, leading to controversy in the field. Specifically, Andre et al. [28] showed a higher GRS in men, while Peng et al. [17] did not observe noticeable sex differences. In contrast, we confirmed that women had a higher GRS on a large sample basis, which was in line with a very recent CMR-FT study [29]. The authors hypothesized that variability in demographic factors and sample sizes might be the main reasons for these inconsistencies. Regarding the RV, we demonstrated that the magnitude of GLS was greater in women, which is consistent with most previous studies [19, 30, 31]. Additionally, we found that the amplitudes of both the RV GCS and GRS were greater in women, which is in line with some prior CMR-FT studies [18, 32]. Of note, we also confirmed significant sex differences in regional myocardial deformation parameters, which manifested as women having larger absolute biventricular radial and circumferential strain measurements at the basal, mid-cavity, and apical levels than men.

At present, little data are available regarding the associations of biventricular SR with sex. For the LV, we first confirmed that the magnitudes of radial, circumferential, and longitudinal PSSR and PDSR were all greater in women. For the RV, most previous studies merely focused on the association of sex and longitudinal SR, and the conclusions were not always consistent. For example, Liu et al. found that there were no sex-related differences in PSSRL or PDSRL [33], while Qu et al. [19] reported that sex was correlated only with PDSRL and had no significant correlation with PSSRL. With this study, we have added to the body of literature with data derived from a large sample showing greater amplitudes of PSSRL and PDSRL in women.

### Correlation between age and biventricular strain and SR

Many studies have investigated the correlation between myocardial strain and age. For the LV, most studies agree that there is an age-related increase in GRS, while there is no age-dependent variation in GLS [17, 28, 34], which is in agreement with our findings. The relationship between GCS scores and age is widely variable in the recent literature. For example, some studies confirmed that GCS became less negative [17] or remained unchanged [28] with advancing age. In contrast, we observed that aging was associated with an increased amplitude of GCS, which concurred with the findings of some previous studies [34–36]. For the RV, some recent studies have shown that age is significantly associated with GLS only in women [18, 31]. In the present study, we observed a linear correlation between age and GLS in both sexes, showing that aging was associated with an increase in the absolute value of GLS. Furthermore, we confirmed an increase in the magnitude of RV GRS and GCS those with aging, which is in accordance with the study by Liu et al. [18]. To date, the correlation between age and biventricular regional deformation indices has not been well explored. We observed larger magnitudes of both LV and RV radial and circumferential strain at the basal, mid-cavity, and apical levels with aging, which paralleled the global measurements in their corresponding directions.

The relationship between aging and biventricular SR has not been well clarified. The present study demonstrated that aging was not significantly correlated with LV PSSR, while it was related to an increase in RV PSSR. Concerning the relationship between aging and myocardial PDSR, Andre et al. [28] showed an age-dependency for the PDSRR, PDSRC, and PDSRL of the LV, which was consistent with our results. For the RV, consistent with the study by Liu et al. [18], we have also shown that aging was associated with PDSRR and PDSRC but not with PDSRL.

### Reproducibility

Reproducible assessment of cardiac function is essential in clinical routine. In agreement with previous publications [18, 29], the present study demonstrated good to excellent intra- and interobserver reproducibility in the measurements of biventricular strain. In addition, we found that the measurements for the LV had slightly higher reproducibility than those for the RV, which might be due to the relatively thin wall and complex structure of the RV.

### Limitations

This study has several limitations. First, normative values for adults over 70 years old were not obtained due to the limited number of elderly individuals who met our strict inclusion criteria. However, the healthy subjects in this study were selected following strict criteria and the sample size was large enough to represent a reasonable sample, so our findings are worthy of generalization. Second, our results cannot be extended to ethnic groups other than Asians peoples because different ethnic groups have different values of myocardial deformation. For the same reasons, this may be one of the biggest advantages of our research because the variability related to race is greatly reduced. Finally, there may be a socioeconomic bias in the sample selection as the subjects included were those able to afford the extensive health screening.

## Conclusions

We provide age- and sex-specific reference values of biventricular strain and SR measured by CMR-FT in a large sample of healthy Chinese adults with a broad age range. In addition, we showed that biventricular deformation parameters are significantly affected by age and sex, adding to the body of knowledge in the field. The present findings may be used as a reference standard for CMR evaluation of cardiac function, especially for the Chinese population.

## Supplementary Information

**Additional file 1: Fig. S1.** Example of biventricular myocardial deformation analysis by Medis (Medis Medical Imaging, Leiden, the Netherlands)**.** Contours are illustrated in LV endocardial and epicardial borders in short-axis view (**a**), four-chamber view (**b**), three-chamber view (**c**), and two-chamber view (**d**), and RV endocardial in four-chamber view (**e**).

**Additional file 2: Fig. S2.** Correlation between biventricular global strain and age assessed by Medis. Linear regressions with the 95% prediction band for LV global radial strain (GRS) (**a**), global circumferential strain (GCS) (**b**), global longitudinal strain (GLS) (**c**), and RV free wall- global longitudinal strain (RVFW-GLS) (**d**).

**Additional file 3: Table S1.** Normal values of biventricular global strain for men and women measured by Medis. **Table S2.** Biventricular strain by age decades for men measured by Medis (n = 50). **Table S3.** Biventricular strain by age decades for women measured by Medis (n = 50). **Table S4.** Comparison of left ventricular global strain measured by cvi^42^ and Medis (n = 100). **Table S5.** Comparison of biventricular strain based on the two MRI scanners (n = 20). **Table S6.** Reproducibility of manual and automatic contouring methods of LV and RV global strain assessed by cvi^42^ (n = 60). **Table S7.** Intra- and interobserver reproducibility of LV global strain and RVFW stain parameters assessed by Medis (n = 60). **Table S8.** Reproducibility of biventricular strain metrics by the two MRI scanners (n = 20).

## Data Availability

The datasets used and/or analyzed in the current study are available from the corresponding author on reasonable request.

## Abbreviations

ANOVA: Analysis of variance
BMI: Body mass index
BSA: Body surface area
bSSFP: Balanced steady state free precession
CI: Confidence interval
CMR: Cardiovascular magnetic resonance
CMR-FT: Cardiovascular magnetic resonance-feature tracking
DBP: Diastolic blood pressure
DENSE: Displacement encoding with stimulated echoes
ECG: Electrocardiogram
FBG: Fasting blood glucose
FT: Feature tracking
GCS: Global circumferential strain
GLS: Global longitudinal strain
GRS: Global radial strain
HR: Heart rate
ICC: Intra-class correlation coefficient
LV: Left ventricle/left ventricular
PDSR: Peak diastolic strain rate
PDSRC: Peak diastolic strain rate circumferential
PDSRL: Peak diastolic strain rate longitudinal
PDSRR: Peak diastolic strain rate radial
PSSR: Peak systolic strain rate
PSSRC: Peak systolic strain rate circumferential
PSSRL: Peak systolic strain rate longitudinal
PSSRR: Peak systolic strain rate radial
RV: Right ventricle/right ventricular
RVFW: Right ventricular free wall
SBP: Systolic blood pressure
SENC: Strain encoding
SR: Strain rate
