# Supplementary figures and images for: Age- and sex-specific reference values of biventricular strain and strain rate derived from a large cohort of healthy Chinese adults: a cardiovascular magnetic resonance feature tracking study
Source: J Cardiovasc Magn Reson. 2022 Nov 21;24:63. doi: 10.1186/s12968-022-00881-1 (PMC9677678; doi:10.1186/s12968-022-00881-1)

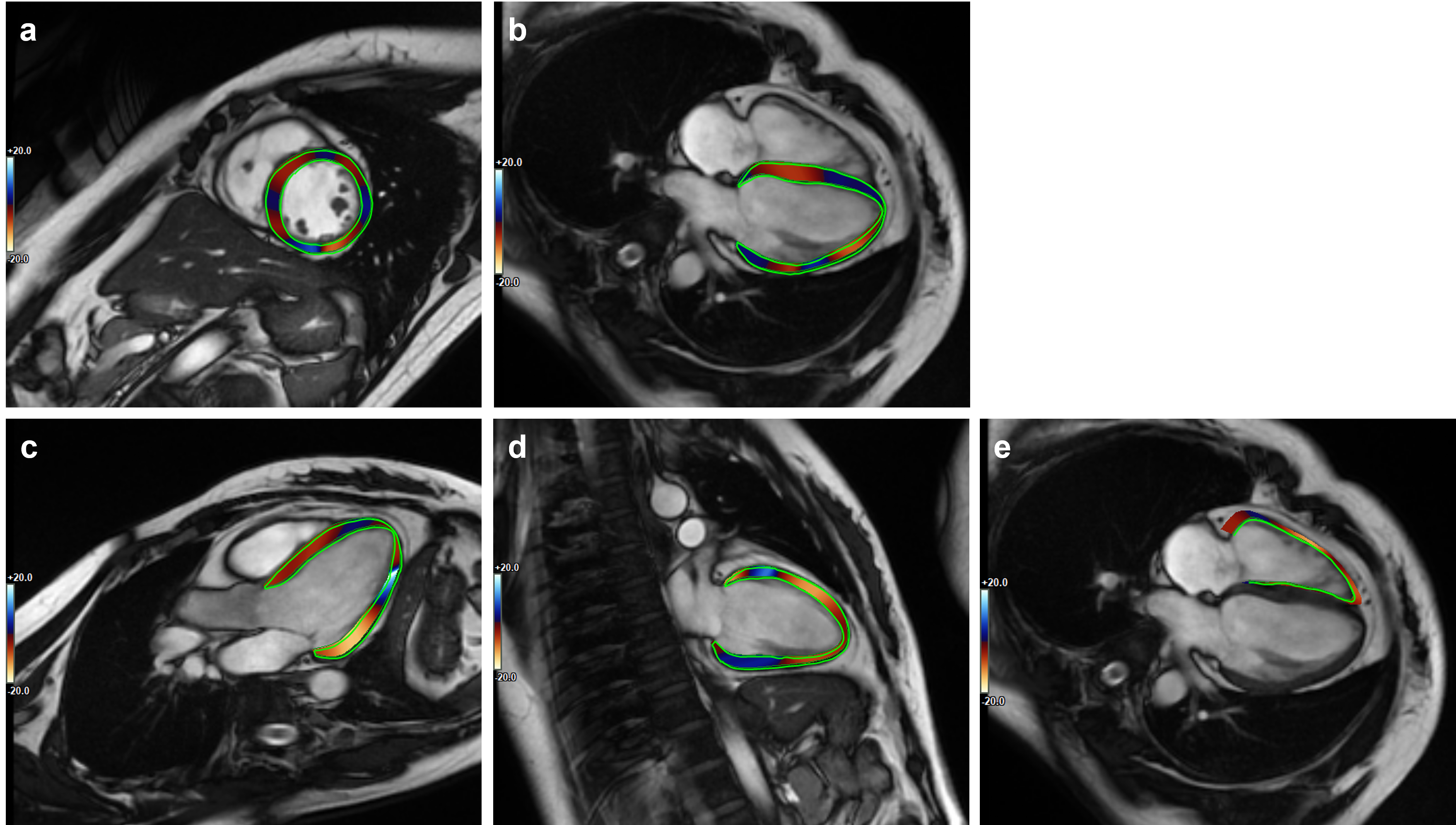

Supplement: Supplementary file 1 — Additional file 1: Fig. S1. Example of biventricular myocardial deformation analysis by Medis (Medis Medical Imaging, Leiden, the Netherlands). Contours are illustrated in LV endocardial and epicardial borders in short-axis view (a), four-chamber view (b), three-chamber view (c), and two-chamber view (d), and RV endocardial in four-chamber view (e). [file 12968_2022_881_MOESM1_ESM.tif]
