# Supplementary material for: Age- and sex-specific reference values of biventricular strain and strain rate derived from a large cohort of healthy Chinese adults: a cardiovascular magnetic resonance feature tracking study
Source: J Cardiovasc Magn Reson. 2022 Nov 21;24:63. doi: 10.1186/s12968-022-00881-1 (PMC9677678; doi:10.1186/s12968-022-00881-1)
Supplement: Supplementary file 3 — Additional file 3: Table S1. Normal values of biventricular global strain for men and women measured by Medis. Table S2. Biventricular strain by age decades for men measured by Medis (n = 50). Table S3. Biventricular strain by age decades for women measured by Medis (n = 50). Table S4. Comparison of left ventricular global strain measured by cvi42 and Medis (n = 100). Table S5. Comparison of biventricular strain based on the two MRI scanners (n = 20). Table S6. Reproducibility of manual and automatic contouring methods of LV and RV global strain assessed by cvi42 (n = 60). Table S7. Intra- and interobserver reproducibility of LV global strain and RVFW stain parameters assessed by Medis (n = 60). Table S8. Reproducibility of biventricular strain metrics by the two MRI scanners (n = 20). [file 12968_2022_881_MOESM3_ESM.docx]

| **Table S1.** Normal values of biventricular global strain for men and women measured by Medis | | | | |
| --- | --- | --- | --- | --- |
| Variables | All (n = 100) | Men (n = 50) | Women (n = 50) | *P* Value (gender) |
| LVGRS (%) | 51.4 ± 17.3 | 51.8 ± 15.4 | 51.0 ± 19.2 | 0.808 |
| LVGCS (%) | -22.2 ± 3.4 | -20.8 ± 2.9 | -23.6 ± 3.3 | ＜0.001 |
| LVGLS (%) | -22.6 ± 3.7 | -20.8 ± 2.4 | -24.4 ± 3.9 | ＜0.001 |
| RVFW-GLS (%) | -26.6 ± 5.9 | -24.2 ± 5.7 | -28.9 ± 5.1 | ＜0.001 |
| Data are presented as means ± standard deviation.  GCS, global circumferential strain; GLS, global longitudinal strain; GRS, global radial strain; LV, left ventricular; RVFW, right ventricular free wall. | | | | |

| **Table S2.** Biventricular strain by age decades for men measured by Medis (n = 50) | | | | | |
| --- | --- | --- | --- | --- | --- |
| Variables | 21-30 years  (n = 10) | 31-40 years  (n = 10) | 41-50 years  (n = 10) | 51-60 years  (n = 10) | 61-70 years  (n = 10) |
| LVGRS (%) | 46.2 ± 8.6 | 50.5 ± 16.9 | 52.5 ± 19.4 | 50.5 ± 16.5 | 59.5 ± 13.1 |
| LVGCS (%) | -20.4 ± 3.3 | -19.2 ± 1.9 | -20.5 ± 2.7 | -21.9 ± 3.5 | -22.1 ± 2.3 |
| LVGLS (%) | -21.1 ± 3.6 | -19.6 ± 1.6 | -21.1 ± 1.9 | -21.2 ± 2.1 | -20.9 ± 2.3 |
| RVFW-GLS (%) | -23.3 ± 3.5 | -20.1 ± 5.2 | -25.6 ± 5.3 | -25.5 ± 5.8 | -26.7 ± 6.8 |
| Data are presented as means ± standard deviation.  GCS, global circumferential strain; GLS, global longitudinal strain; GRS, global radial strain; LV, left ventricular; RVFW, right ventricular free wall. | | | | | |

| **Table S3.** Biventricular strain by age decades for women measured by Medis (n = 50) | | | | | |
| --- | --- | --- | --- | --- | --- |
| Variables | 21-30 years  (n = 10) | 31-40 years  (n = 10) | 41-50 years  (n = 10) | 51-60 years  (n = 10) | 61-70 years  (n = 10) |
| LVGRS (%) | 41.6 ± 11.5 | 43.6 ± 17.3 | 50.5 ± 22.4 | 57.4 ± 14.5 | 61.8 ± 23.1 |
| LVGCS (%) | -21.9 ± 2.0 | -21.9 ± 4.2 | -24.3 ± 1.4 | -25.3 ± 3.2 | -24.6 ± 3.3 |
| LVGLS (%) | -25.2 ± 2.2 | -24.1 ± 2.6 | -25.1 ± 1.5 | -22.6 ± 6.5 | -25.1 ± 4.3 |
| RVFWGLS (%) | -27.3 ± 4.5 | -28.1 ± 3.5 | -27.2 ± 3.7 | -30.0 ± 5.4 | -31.8 ± 7.0 |
| Data are presented as means ± standard deviation.  GCS, global circumferential strain; GLS, global longitudinal strain; GRS, global radial strain; LV, left ventricular; RVFW, right ventricular free wall. | | | | | |

| **Table S4** Comparison of left ventricular global strain measured by cvi42 and Medis (n = 100) | | | |
| --- | --- | --- | --- |
|  | cvi42 | Medis | *P* Value |
| GRS (%) | 35.5 ± 7.0 | 51.4 ± 17.3 | 0.002 |
| GCS (%) | -19.9 ± 2.3 | -22.2 ± 3.4 | <0.001 |
| GLS (%) | -16.8 ± 2.2 | -22.6 ± 3.7 | <0.001 |

Data are presented as means ± standard deviation.

GRS, global peak radial strain; GLS, global peak longitudinal strain; GCS, global peak circumferential strain.

| **Table S5** Comparison of biventricular strain based on the two MRI scanners (n = 20) | | | |
| --- | --- | --- | --- |
| Variables | GE | Siemens | *P* Value |
| LV GRS (%) | 32.0 ± 4.6 | 32.1 ± 4.5 | 0.818 |
| LV GCS (%) | -18.6 ± 1.5 | -18.8 ± 1.5 | 0.332 |
| LV GLS (%) | -17.8 ± 2.0 | -17.6 ± 2.0 | 0.252 |
| RV GRS (%) | 24.7 ± 6.2 | 23.9 ± 5.4 | 0.149 |
| RV GCS (%) | -14.7 ± 3.2 | -14.5 ± 2.6 | 0.509 |
| RV GLS (%) | -26.4 ± 2.0 | -26.3 ± 2.1 | 0.697 |

Data are presented as means ± standard deviation. The *P* Value is for a paired sample *t* test between the two MRI scanners.

GCS, global peak circumferential strain; GLS, global peak longitudinal strain; GRS, global peak radial strain; LV, left ventricular; RV, right ventricular.

| **Table S6** Reproducibility of manual and automatic contouring methods of LV and RV global strain assessed by cvi42 (n = 60) | | | |
| --- | --- | --- | --- |
| Variables | Mean difference ± SD | Limits of agreement | ICC (95% CI) |
| LVGRS (%) | 2.76 ± 1.93 | -1.02 to 6.53 | 0.94 (0.90,0.96) |
| LVGCS (%) | 2.69 ± 1.95 | -1.13 to 6.53 | 0.94 (0.91,0.97) |
| LVGLS (%) | -0.14 ± 0.89 | -1.88 to 1.60 | 0.93 (0.88,0.95) |
| RVGRS (%) | -0.34 ± 3.26 | -6.73 to 6.06 | 0.90 (0.84,0.94) |
| RVGCS (%) | 0.34 ± 1.89 | -3.35 to 4.05 | 0.86 (0.78,0.91) |
| RVGLS (%) | -0.51 ± 2.17 | -4.77 to 3.75 | 0.90 (0.84,0.94) |

CI, confidence interval; ICC, intra-class correlation coefficient; GCS, global peak circumferential strain; GLS, global peak longitudinal strain; GRS, global peak radial strain; LV, left ventricular; RV, right ventricular; SD, standard deviation.

| **Table S7.** Intra- and interobserver reproducibility of LV global strain and RVFW stain parameters assessed by Medis (n = 60) | | | | |
| --- | --- | --- | --- | --- |
| Variables | Variability | Mean absolute bias | Limits of agreement | ICC (95% CI) |
| LVGRS (%) | Intraobserver | 0.21 ± 3.95 | -7.54 to 7.96 | 0.97 (0.95, 0.98) |
|  | Interobserver | 3.80 ± 7.42 | -10.75 to 18.35 | 0.86 (0.72, 0.93) |
| LVGCS (%) | Intraobserver | 0.09 ± 1.00 | -1.87 to 2.05 | 0.95 (0.92, 0.97) |
|  | Interobserver | -1.49 ± 1.14 | -3.71 to 0.74 | 0.83 (0.09, 0.95) |
| LVGLS (%) | Intraobserver | 0.02 ± 0.79 | -1.52 to 1.56 | 0.98 (0.96, 0.99) |
|  | Interobserver | 0.65 ± 2.23 | -3.71 to 5.01 | 0.79 (0.67, 0.87) |
| RVFW-GLS (%) | Intraobserver | -0.22 ± 2.54 | -5.20 to 4.76 | 0.92 (0.88, 0.95) |
|  | Interobserver | 0.16 ± 2.80 | -5.33 to 5.65 | 0.90 (0.84, 0.94) |
| LV, left ventricular; RVFW, right ventricular free wall; GRS, global radial strain; GLS, global longitudinal strain; GCS, global circumferential strain; ICC, intra-class correlation coefficient; CI, confidence interval. | | | | |

| **Table S8** Reproducibility of biventricular strain metrics by the two MRI scanners (n = 20) | | | |
| --- | --- | --- | --- |
| Variables | Mean difference ± SD | Limits of agreement | ICC (95% CI) |
| LVGRS (%) | 0.12 ± 2.30 | -4.40 to 4.64 | 0.88 (0.71,0.95) |
| LVGCS (%) | -0.20 ± 0.91 | -1.99 to 1.59 | 0.82 (0.60,0.92) |
| LVGLS (%) | 0.27 ± 1.02 | -1.72 to 2.26 | 0.87 (0.71,0.95) |
| RVGRS (%) | -0.77 ± 2.28 | -5.23 to 3.70 | 0.92 (0.81,0.97) |
| RVGCS (%) | 0.19 ± 1.29 | -2.33 to 2.72 | 0.90 (0.78,0.96) |
| RVGLS (%) | 0.10 ± 1.15 | -2.16 to 2.37 | 0.84 (0.65,0.94) |

Biventricular strain and strain rates were measured by cvi42.

CI, confidence interval; ICC, intra-class correlation coefficient; GCS, global peak circumferential strain; GLS, global peak longitudinal strain; GRS, global peak radial strain; LV, left ventricular; RV, right ventricular; SD, standard deviation.
